# Peripheral-neuron-like properties of differentiated human dental pulp stem cells (hDPSCs)

**DOI:** 10.1371/journal.pone.0251356

**Published:** 2021-05-06

**Authors:** Yuki Arimura, Yutaka Shindo, Ryu Yamanaka, Mai Mochizuki, Kohji Hotta, Taka Nakahara, Etsuro Ito, Tohru Yoshioka, Kotaro Oka

**Affiliations:** 1 Faculty of Science and Technology, Department of Bioscience and Informatics, Keio University, Kanagawa, Japan; 2 Faculty of Pharmaceutical Sciences, Sanyo-Onoda City University, Yamaguchi, Japan; 3 Department of Life Science Dentistry, The Nippon Dental University, Tokyo, Japan; 4 Department of Developmental and Regenerative Dentistry, School of Life Dentistry at Tokyo, The Nippon Dental University, Tokyo, Japan; 5 Waseda Research Institute for Science and Engineering, Waseda University, Tokyo, Japan; 6 Department of Biology, Waseda University, Tokyo, Japan; 7 Graduate Institute of Medicine, College of Medicine, Kaohsiung Medical University, Kaohsiung, Taiwan; MAHSA University, MALAYSIA

## Abstract

Elucidating the mechanisms underlying human pain sensation requires the establishment of an *in vitro* model of pain reception comprising human cells expressing pain-sensing receptors and function properly as neurons. Human dental pulp stem cells (hDPSCs) are mesenchymal stem cells and a promising candidate for producing human neuronal cells, however, the functional properties of differentiated hDPSCs have not yet been fully characterized. In this study, we demonstrated neuronal differentiation of hDPSCs via both their expression of neuronal marker proteins and their neuronal function examined using Ca^2+^ imaging. Moreover, to confirm the ability of nociception, Ca^2+^ responses in differentiated hDPSCs were compared to those of rat dorsal root ganglion (DRG) neurons. Those cells showed similar responses to glutamate, ATP and agonists of transient receptor potential (TRP) channels. Since TRP channels are implicated in nociception, differentiated hDPSCs provide a useful *in vitro* model of human peripheral neuron response to stimuli interpreted as pain.

## Introduction

The peripheral nervous system (PNS) receives environmental information. Pain perception is an important function that allows for the detection of noxious stimuli in order to protect the body. In addition, relief of chronic pain is an important component of improving quality of life [1]. Noxious stimuli are detected by skin tissue and the PNS, and the nociceptive signals they produce are transmitted to the central nervous system (CNS), where they are recognized as pain [2–4]. To elucidate the mechanisms of acute pain perception, it is important to understand the sensation mechanisms present in skin cells and peripheral nerves, as well as the information processing and modulation that take place in the PNS [5]. While experiments in model animals and in cultured dorsal root ganglion (DRG) neurons from mice and rats have provided important insight into pain reactions [6–8], it is also required to establish an adequate *in vitro* model of pain receptions in peripheral systems composed of human cells. To achieve this, it is necessary to prepare human cells that have neuronal functions and express receptors to receive nociceptive stimuli [9,10].

Although it is difficult to extract human peripheral neurons for experimental use, with stem cells it is possible to establish cultured human tissues and neurons *in vitro*. Embryonic stem (ES) cells and induced pluripotent stem (iPS) cells have the potential to differentiate toward most cell types in the body [11,12], and it has been shown that these cells also have the potential to differentiate into peripheral neurons that can then be used in research on nociception [13,14]. Despite the versatility of these cells, in some cases other kinds of stem cell are more useful: they may not need reprogramming, and they may be easier to obtain, proliferate, and differentiate toward a specific cell type [15,16]. Recent studies have shown that human dental pulp stem cells (hDPSCs), a type of neural crest derived stem cells (NCSCs), are a strong candidate for this type of use [17,18]. NCSCs can be obtained even from adult tissues, including bone marrow, cornea skin and dental pulp [19]. Because NCSCs could be applied to autologous transplantation, it has benefits not only for basic science but also for the purpose of regenerative medicine and cell therapy [20]. In those, hDPSCs are mesenchymal stem cells isolated from dental pulp tissue [21]. They can easily be obtained from extracted teeth and possess a high proliferation ability [22,23]. Furthermore, they show multipotency, and it is easy to induce them to differentiate toward osteoblasts, adipocytes, neuronal cells, or astrocytes by culturing them in the appropriate differentiation medium [24–29]. In spite of their stemness, hDPSCs do not readily form tumors *in vivo* [30], and they show more accurately preserved DNA methylation patterns than ES and iPS cells [31]. They may therefore be a better stem cell source for both disease modeling and regenerative therapies [32,33].

Because many researches demonstrated that hDPSCs have a potential to differentiate toward neurons, hDPSC expected to be used in therapies for neurodegenerative diseases and in fundamental studies of human neural systems [32]. Expression of neuronal marker proteins has been confirmed in neuronal differentiated hDPSCs [26,34]. Like conventional neurons, they possess functional and active voltage-gated sodium and potassium channels [35,36], and, in some cases, generate action potentials [37]. It has been reported that hDPSCs could differentiate to dopaminergic, glutamatergic and GABAergic neuron-like cells [32]. Differentiated hDPSCs have already been used in cellular and animal models for the study of CNS conditions such as ischemia and neurodegenerative diseases like Parkinson’s and Alzheimer’s diseases [38–41]. These studies indicate that hDPSCs have the potential to differentiate toward neuron-like cells that function in the CNS. On the other hand, some studies have reported that hDPSCs also differentiated toward bipolar shape neuron-like cells similar to peripheral neurons [36,42]. However, whether the differentiated hDPSCs have peripheral neuron-like characteristics have not been investigated. To establish *in vitro* model of human nociception using hDPSCs, it is needed that neuronal differentiated hDPSCs show peripheral neuron-like responses. To confirm this, it is necessary to compare the responsiveness of neuronal differentiated hDPSCs with PNS-derived neurons.

In this study, we observed the responses of differentiated hDPSCs by using Ca^2+^ imaging to characterize the function of these cells by their activity patterns. By comparing the expression of neuronal marker proteins and the Ca^2+^ responses of each cell, we further confirmed the differentiation of hDPSCs toward neuronal cells. We also analyzed the Ca^2+^ responses to stimuli relating to nociception and assessed the potential for using hDPSCs as an *in vitro* model of peripheral neurons by comparing their responses to those of cultured DRG neurons.

## Materials and methods

### Ethical approval

The use of human stem cells was approved by the ethics committees of the Nippon Dental University School of Life Dentistry at Tokyo (permit number: NDU-T2013-10) and the Department of Science and Technology, Keio University (permit numbers: 30–31 and 2020–15). Written informed consent was obtained from all individual donors after fully explaining the nature of the procedure and the intended use of the tissue obtained. All experiments were carried out in accordance with relevant guidelines and regulations (Declaration of Helsinki) in the manuscript.

All animal procedures were approved by the ethics committee of Keio University (permit number: 09106(1)). All methods were carried out in accordance with relevant guidelines and regulations in the manuscript.

### Culture of hDPSCs

Isolation and culturing of hDPSCs were performed as previously described [43,44]. The cells were plated at a density of 1–2 × 10^4^ cells/mL onto glass-bottom dishes (AGC techno glass, Tokyo, Japan). They were cultured in DMEM/F12 (Thermo Fisher Scientific, Waltham, MA, USA) supplemented with 1 mL/L MEM NEAA (Thermo Fisher Scientific), 2.4 g/L NaHCO_3_, 1 mL/L Fungizone (GE Healthcare, Chicago, IL, USA), 500 μL/L GlutaMAX (Thermo Fisher Scientific), 50 U/mL penicillin, and 50 μg/mL streptomycin (Nacalai tesque, Kyoto, Japan). Cultures were maintained in a 5% CO_2_ incubator at 37°C and the culture medium was changed every three days.

hDPSCs were differentiated by culturing them in differentiation medium consisting of DMEM/F12 supplemented with 5% FBS, 10 μM MEM-NEAAs, 2 mM glutamate (Nacalai tesque), 10 nM all trans-retinoic acids (Merck, Darmstadt, Germany), 50 μM ascorbic acid (Tokyo Chemical Industry, Tokyo, Japan), 5 μM insulin (Cell Signaling Technology, Danvers, MA, USA), 10 nM dexamethasone (FUJIFILM, Tokyo, Japan), 20 nM progesterone (Tokyo Chemical Industry), 20 nM estradiol (FUJIFILM), 50 ng/mL nerve growth factor (NGF; Alomone Labs, Jerusalem, Israel), 10 ng/mL thyroxine (T4; FUJIFILM), 50 U/mL penicillin, 50 μg/mL streptomycin, and 0.25 μg/mL Fungizone as described previously [42]. For fluorescence measurements, cells were cultured in glass-bottom dishes with or without grids (AGC techno glass). The medium was changed every three days.

### Dissociation culture of rat DRG neurons and rat hippocampal neurons

DRG were isolated from day 18 embryonic Wister rats (Charles River Laboratories, Wilmington, MA, USA). Cells were extirpated from 4–8 embryos at a time and submerged in ice-cold phosphate-buffered saline (PBS). DRG neurons were dissociated using trypsin and plated at a density of 4 × 10^4^ cells/mL onto poly-D-lysine and laminin (Merck) coated glass-bottom dishes. The neurons were cultured in neurobasal medium containing B-27 (Thermo Fisher Scientific), 50 ng/mL NGF, 50 U/mL penicillin, and 50 μg/mL streptomycin for five days.

Hippocampi were isolated from day 18 embryonic Wister rats and dissociated using Nerve Cell Dissociation Medium (Sumitomo Bakelite, Tokyo, Japan). They were plated at a density of 20 × 10^4^ cells/mL onto poly-D-lysine coated glass bottom dishes. The neurons were cultured in neurobasal medium with B-27, 2 mM glutamine, 50 U/mL penicillin, and 50 μg/mL streptomycin for twelve days.

### Immunofluorescence imaging

Immunofluorescence staining was performed according to the previously described method [45]. Cultured cells were fixed in 4% paraformaldehyde (PFA; Nacalai tesque) diluted in PBS for 20 min at room temperature. Samples were washed twice in PBS to remove residual PFA. They were then permeabilized in PBS with 0.1% Triton X-100 (PBT) for 1 min and incubated in PBT with 10% goat serum for 30 min for blocking. To compare the expression of βIII-tubulin, nestin, and GFAP, mouse anti-βIII-tubulin (1:250, Merck), chicken anti-nestin (1:2000, Abcam, Cambridge, UK) and rabbit anti-GFAP (1:1000, Abcam) were diluted in blocking solution and applied to the samples for 60 min at room temperature. The samples were then washed three times with PBT and incubated in blocking solution containing 4′,6-diamidino-2-phenylindole (DAPI; 1:1000, Dojindo laboratories, Tokyo, Japan) and Alexa Fluor 488 anti-rabbit IgG, Alexa Fluor 546 anti-chicken IgG, and Alexa Fluor 633 anti-mouse IgG (each at 1:1000, Thermo Fisher Scientific) for 40 min at room temperature. The samples were washed three times with PBT and then filled with PBS. To compare the expression of Brn-3a, TRPV1 and substance-P, mouse anti-Brn-3a (1:50, Santa Cruz Biotechnology, Dallas, TX, USA), rabbit anti-TRPV1 (1:250, Abcam) and rat anti-substance-P (1:100, Abcam) were used as primary antibodies, and the samples were stained with secondary antibodies, Alexa Fluor 488 anti-mouse IgG, Alexa Fluor 546 anti-rabbit IgG, Alexa Fluor 633 anti-rat IgG (each at 1:1000, Thermo Fisher Scientific), and DAPI.

To compare the expression of Brn-3a and substance-P after Ca^2+^ imaging (described below), the samples fixed immediately after the Ca^2+^ imaging were probed with mouse anti-Brn-3a and rat anti-substance-P and stained with Alexa Fluor 405 anti-mouse IgG and Alexa Fluor 633 anti-rat IgG. To compare the expression of βIII-tubulin and GFAP after Ca^2+^ imaging (described below), the samples fixed immediately after the Ca^2+^ imaging were probed with mouse anti-βIII-tubulin and rabbit anti-GFAP and stained with Alexa Fluor 546 anti-mouse IgG and Alexa Fluor 633 anti-rabbit IgG.

**Fig 1:**
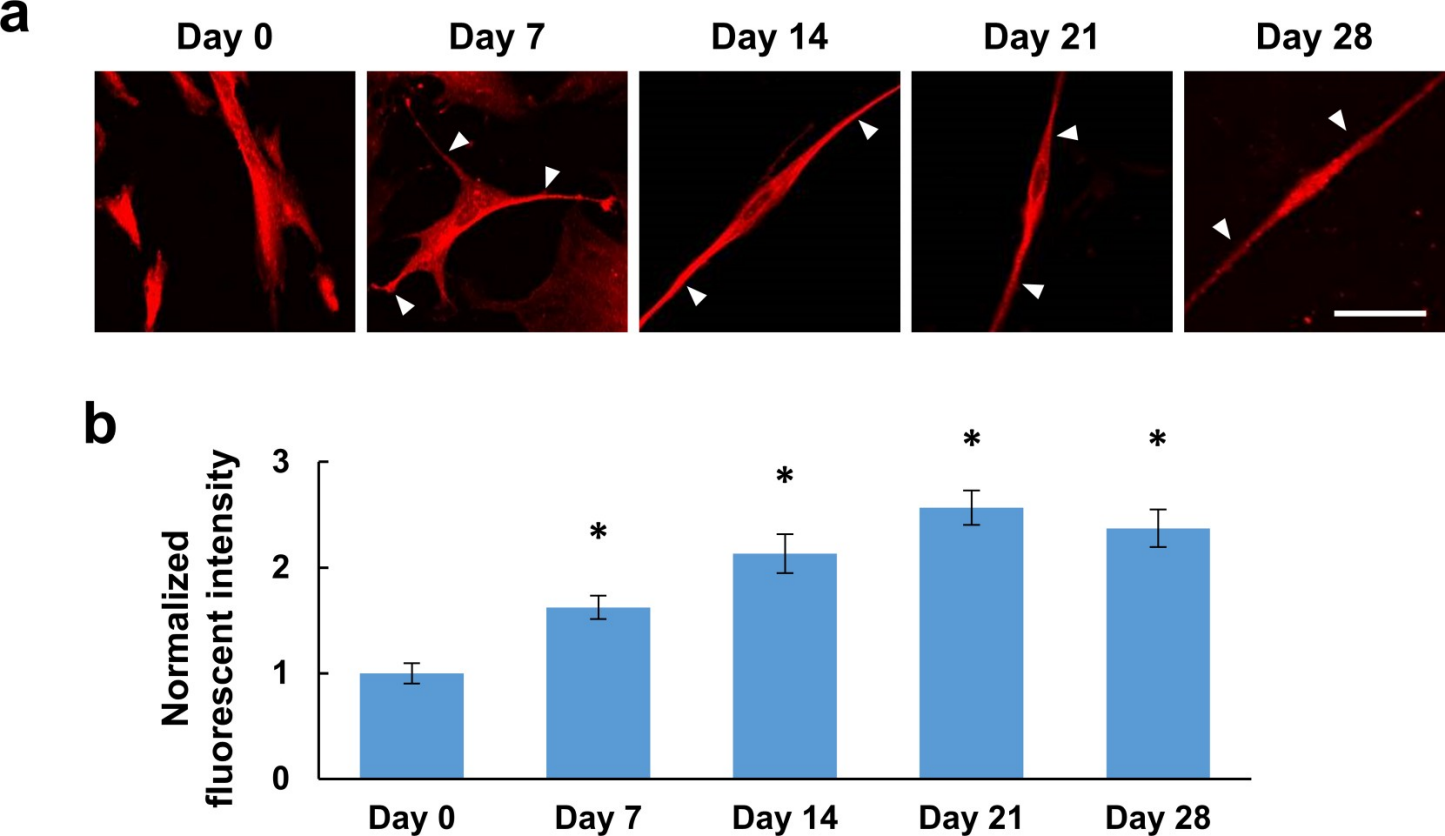
Immunofluorescence characterization of the neural differentiation of human dental pulp stem cells (hDPSCs). (a) Representative confocal immunofluorescent images showing βIII-tubulin of hDPSCs cultured in differentiation medium for 0, 7, 14, 21 or 28 days (Day 0, Day 7, Day 14, Day21 and Day28). Approximately similar changes in cell shape and βIII-tubulin expression levels were shown in 4–5 different experiments for each differentiation stage. Arrowheads in the merged images indicate βIII-tubulin-positive processes. Scale bar, 10 μm. (b) Changes in expression levels of βIII-tubulin during the differentiation process (n = 124, 131, 63, 100, or 72 cells from 4–5 different dishes per differentiation stage). The values were normalized by the averaged fluorescence intensity at Day 0. * *P* < 0.05 compared to Day 0. Error bars, standard error of the mean.

**Fig 2:**
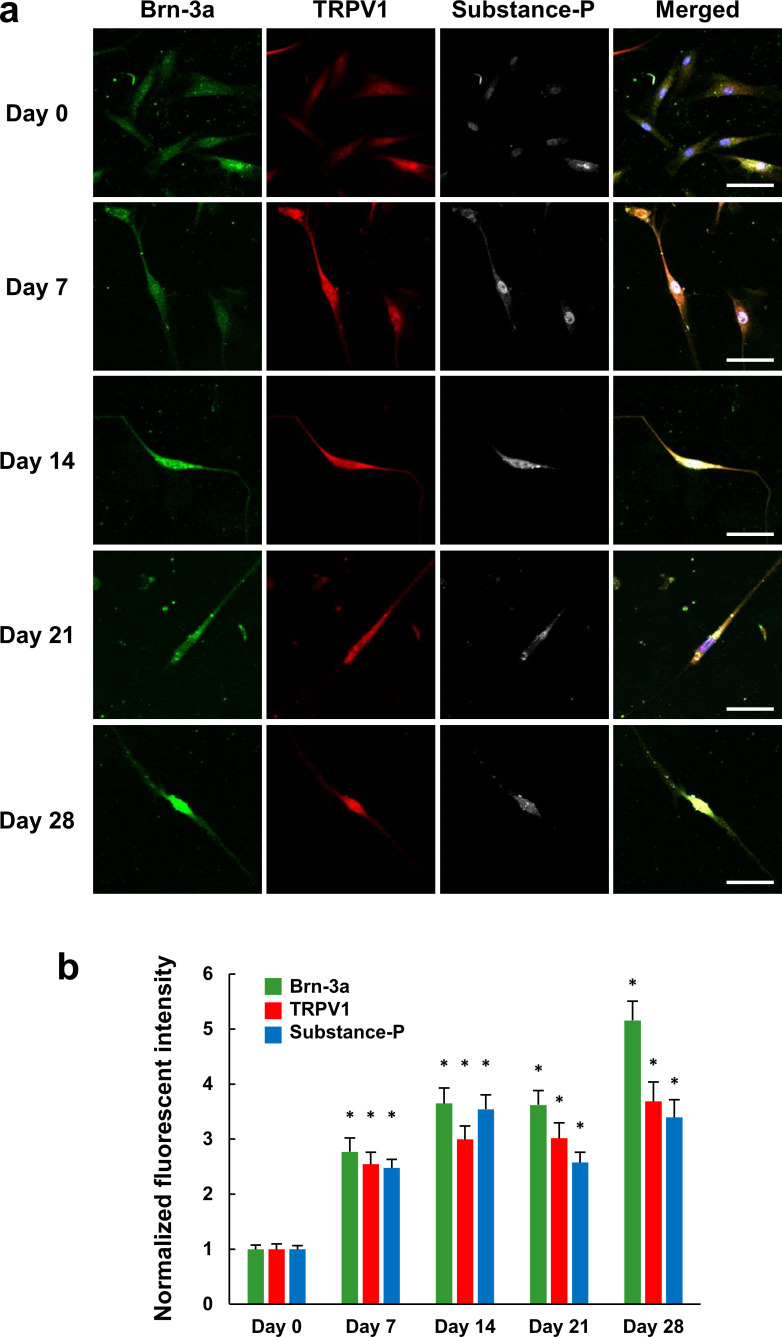
Immunofluorescence characterization of the neural differentiation of hDPSCs by peripheral neuron markers. (a) Confocal immunofluorescent images showing Brn-3a (green), TRPV1 (red), substance-P (white), and merged (DAPI: Blue, Brn-3a: Green, TRPV1: Red, substance-P: White) images of hDPSCs cultured in differentiation medium for 0, 7, 14, 21 or 28 days (Day 0, Day 7, Day 14, Day21 and Day28). Approximately similar changes in the expression levels were shown in 3–4 different experiments for each differentiation stage. Scale bar, 10 μm. (b) Changes in expression levels of Brn-3a, TRPV1, and substance-P during the differentiation process (n = 52, 53, 50, 52, or 48 cells from 3–4 different dishes per differentiation stage, respectively). The values were normalized by the averaged fluorescence intensity at Day 0 for each protein. * *P* < 0.05 compared to Day 0 for each protein. Error bars, standard error of the mean.

**Fig 3:**
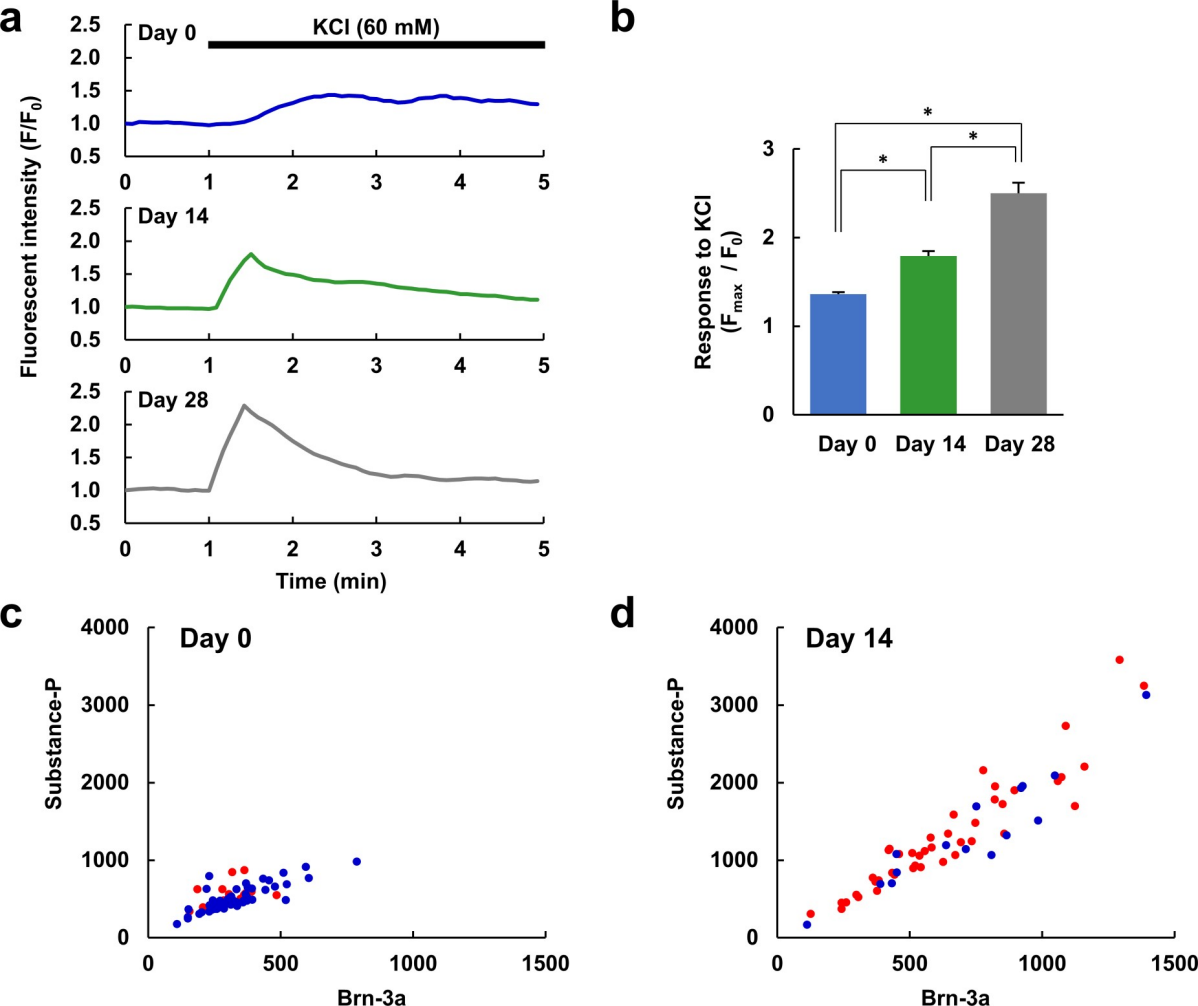
Relationship between neuronal marker expression and Ca^2+^ response. (a) Representative Ca^2+^ responses to high concentration of KCl (60 mM) in undifferentiated hDPSCs (Day 0, upper) and hDPSCs differentiated for 14 days (Day 14, middle) or 28 days (Day 28, bottom). (b) Comparison of the amplitude (F_max_/F_0_) of Ca^2+^ responses evoked by KCl in 1 min after application in Day 0, 14 and 28 hDPSCs (n = 69, 58, or 67 cells, respectively, from 3 different experiments for each condition). * *P* < 0.05 between the indicated pairs. The cells were fixed immediately after Ca^2+^ imaging and immunostained. (c, d) Immunofluorescence intensity of substance-P and Brn-3a of each cell cultured in differentiation medium for 0 days (Day 0, n = 69 cells) (c) and 14 days (Day 14, n = 58 cells) (d). In panels c and g, red dots indicate cells that exhibited a large response to KCl (F_max_/F_0_ > 1.5); blue dots indicate cells that exhibited a small or no response to KCl (F_max_/F_0_ < 1.5).

The samples were observed using a confocal laser scanning microscope system (FluoViewFV1000, OLYMPUS, Tokyo, Japan) mounted on an inverted microscope with ×20 or ×40 oil immersion objectives. DAPI or Alexa Fluor 405, Alexa Fluor 488, Alexa Fluor 546, and Alexa Fluor 633 were sequentially excited with lasers at 405 nm, 488 nm, 559 nm and 635 nm, respectively, and the fluorescence was observed at 425–475 nm for DAPI, 500–545 nm for Alexa Fluor 488, 575–620 nm for Alexa Fluor 546, and 655–755 nm for Alexa Fluor 633. To estimate differentiation-dependent changes in protein expression levels (Figs 1 and 2), we summed the fluorescence intensities in a defined region of interest (ROI) containing the whole cell body of each cell, and then compared averaged values for all cells in each condition. To estimate the expression ratio of Brn-3a to substance-P or βIII-tubulin to GFAP in each cell (Fig 3), averaged fluorescence intensities in each ROI were used.

### Ca^2+^ imaging

For Ca^2+^ imaging, cells were incubated with 5 μM fluo-4 AM (Thermo Fisher Scientific) for 45 min at 37°C. After incubation, the cells were washed twice with Hanks’ Balanced Salt Solution (HBSS, Thermo Fisher Scientific, buffered by 10 mM HEPES and with pH adjusted to 7.4 using NaOH), and then further incubated for 15 min at 37°C. The fluorescence of fluo-4 was measured using a confocal laser scanning microscope system (FluoViewFV1000, OLYMPUS). Fluo-4 was excited at 488 nm through a dichroic mirror (405/488), and fluorescence emission was observed at 500–600 nm. For time lapse imaging, images were obtained every 5 s.

Changes in the fluorescence of each cell were calculated as the mean intensity over a defined ROI containing the cell body. The fluorescence data for each cell were analyzed using MATLAB (MathWorks, Natick, MA, USA). The fluorescence intensity (F) time-courses were normalized by the initial fluorescence (F_0_) of each cell.

Response half-time was defined as the period during which the relative fluorescence intensity (F/F_0_) remained higher than the half-maximum of the intensity of the Ca^2+^ transient evoked by agonists of transient receptor potential (TRP) channels. Cells in which the fluorescence did not drop below the half-maximum of the intensity of the Ca^2+^ transient by the end of the imaging period were eliminated from the analysis because their response half-time could not be calculated.

### Statistics

Significant differences were determined using *t*-tests and the levels of significance were adjusted using Bonferroni correction.

## Results

### Differentiation toward neurons in terms of marker protein expression

After they had been cultured in differentiation medium, the differentiation of hDPSCs toward neurons was confirmed based on the expression levels of βIII-tubulin, a neuron marker (Fig 1). In addition to the increase in expression levels with duration of culturing in differentiation medium, from day 7, some hDPSCs had elongated βIII-tubulin-positive neurite-like processes (arrowheads in Fig 1A). After day 14, some cells showed bipolar type neuron-like shapes (Fig 1A Day 14–28). We also checked the expression levels of βIII-tubulin during the differentiation process (Fig 1B) and confirmed the increase of its expression along the developmental stages and saturation within Day 21.

To further elucidate that hDPSCs differentiated toward peripheral neuron-like calls, expression of Brn-3a, marker for peripheral neurons and retinal ganglion cells, TRPV1, one of the nociceptors, and substance-P, a pain-mediating neurotransmitter, were examined. While undifferentiated hDPSCs (Day 0) slightly expressed all three proteins, their expression levels were significantly increased by differentiation (Fig 2). In addition, while few cells elongated neurite-like processes on Day 0 (3/52 cell, 5.8%), the proportion of cells with elongated processes also increased with differentiation (Day 7: 20/53 cells, 37.7%; Day 14: 32/50 cells, 64.0%; Day 21: 32/52 cells, 61.5%; Day 28: 27/48 cells, 56.3%). Both marker expression and processes elongation appeared to be saturated, in some extent, at Day 14. These results confirm that the hDPSCs differentiated and matured toward peripheral neuronal cells at least in expression levels of marker proteins as a result of being cultured in this medium for 14 days.

### Differentiation toward neurons in terms of function, as revealed by Ca^2+^ imaging

Whether the differentiated cells that expressed neuronal marker protein patterns also showed characteristic neuronal functions was still in question. To assess the neuron-like function in hDPSCs, Ca^2+^ responses to high concentrations of KCl were observed. Conventional neurons maintain their resting membrane potential via the equilibrium potential of K^+^, so an increase in the extracellular K^+^ concentration depolarizes the membrane potential, and when this depolarization surpasses the excitation threshold, voltage-gated Na^+^ and Ca^2+^ channels open. Therefore, if the differentiated hDPSCs express neuron-like ion channels, application of a high concentration of KCl should induce a large increase in their intracellular Ca^2+^ concentration. While KCl induced small and gradual increase in Ca^2+^ in undifferentiated hDPSCs (Day 0), the Ca^2+^ responses became large, steep and transient with neuronal differentiation (Fig 3A and 3B). These data suggest that hDPSCs upregulated not only Ca^2+^ channel but also Ca^2+^ extrusion and storage mechanisms, and that maturation of excitatory Ca^2+^ regulation took more than 28 days.

To evaluate the relationship between the hDPSCs’ expression of marker proteins and their function, cells were fixed immediately after Ca^2+^ imaging, and expression levels of the marker proteins in each cell were evaluated by immunofluorescence. The expression levels of Brn-3a and substance-P in each undifferentiated (Day 0) and differentiated (Day 14) hDPSC were plotted (Fig 3C and 3D). The cells were divided into two groups according to their Ca^2+^ response: large Ca^2+^ response (red; F_max_/F_0_ > 1.5), or small or no Ca^2+^ response (blue; F_max_/F_0_ < 1.5). Most of the undifferentiated cells exhibited a small or no Ca^2+^ response. Whereas both Ca^2+^ responses and expression levels of both proteins were increased with differentiation, the distribution of the plots for large Ca^2+^ response cells and that for small or no Ca^2+^ response cells were almost the same (Fig 3C and 3D).

**Fig 4:**
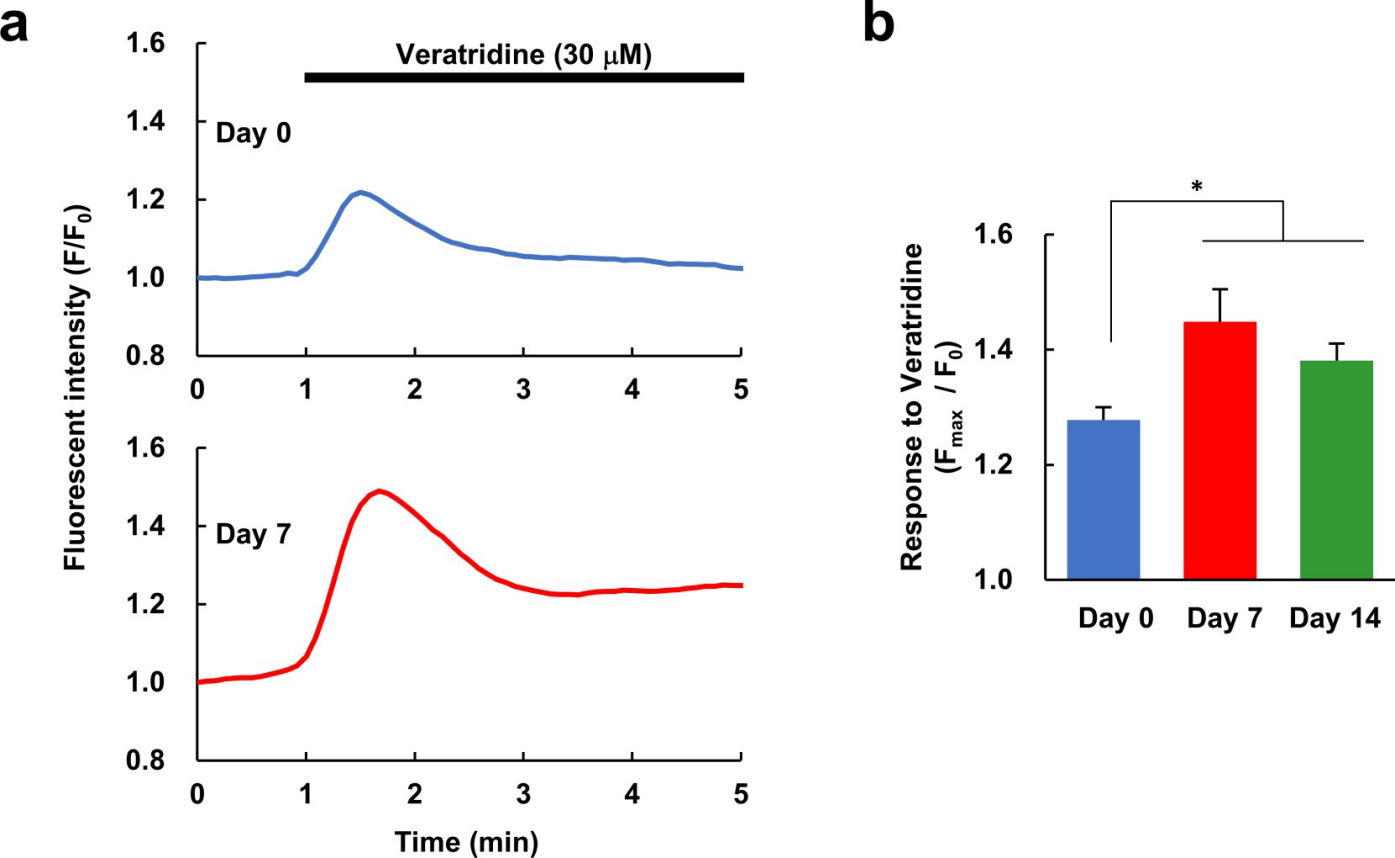
Ca^2+^ response to the voltage-gated Na^+^ channel activator veratridine. (a) Representative Ca^2+^ responses to veratridine (30 μM), an activator of voltage-gated Na^+^ channels, in undifferentiated (Day 0) and differentiated (Day 7) hDPSCs. (b) Comparison of the amplitude (F_max_/F_0_) of Ca^2+^ responses evoked by veratridine in Day 0, 7 and 14 hDPSCs (n = 180, 152, or 84 cells, respectively, from 5–8 different experiments for each condition). Error bars, standard error of the mean. * *P* < 0.05 compared to Day 0.

To further confirm the differentiation of hDPSCs in terms of neuronal function, the expression of voltage-gated Na^+^ channels was analyzed by Ca^2+^ imaging. Veratridine, an activator of voltage-gated Na^+^ channels, elicited a small Ca^2+^ response in undifferentiated (Day 0) hDPSCs, but in differentiated cells (Day 7 and Day 14), the amplitude of the Ca^2+^ response was significantly greater (Fig 4). This indicates that hDPSCs increase their expression of Na^+^ channels during the first seven days of differentiation toward neurons. From these data, we can therefore confirm the differentiation of hDPSCs toward neurons based not only on the expression levels of marker proteins, but also on neuronal functions.

### Ca^2+^ response to other extracellular signals

**Fig 5:**
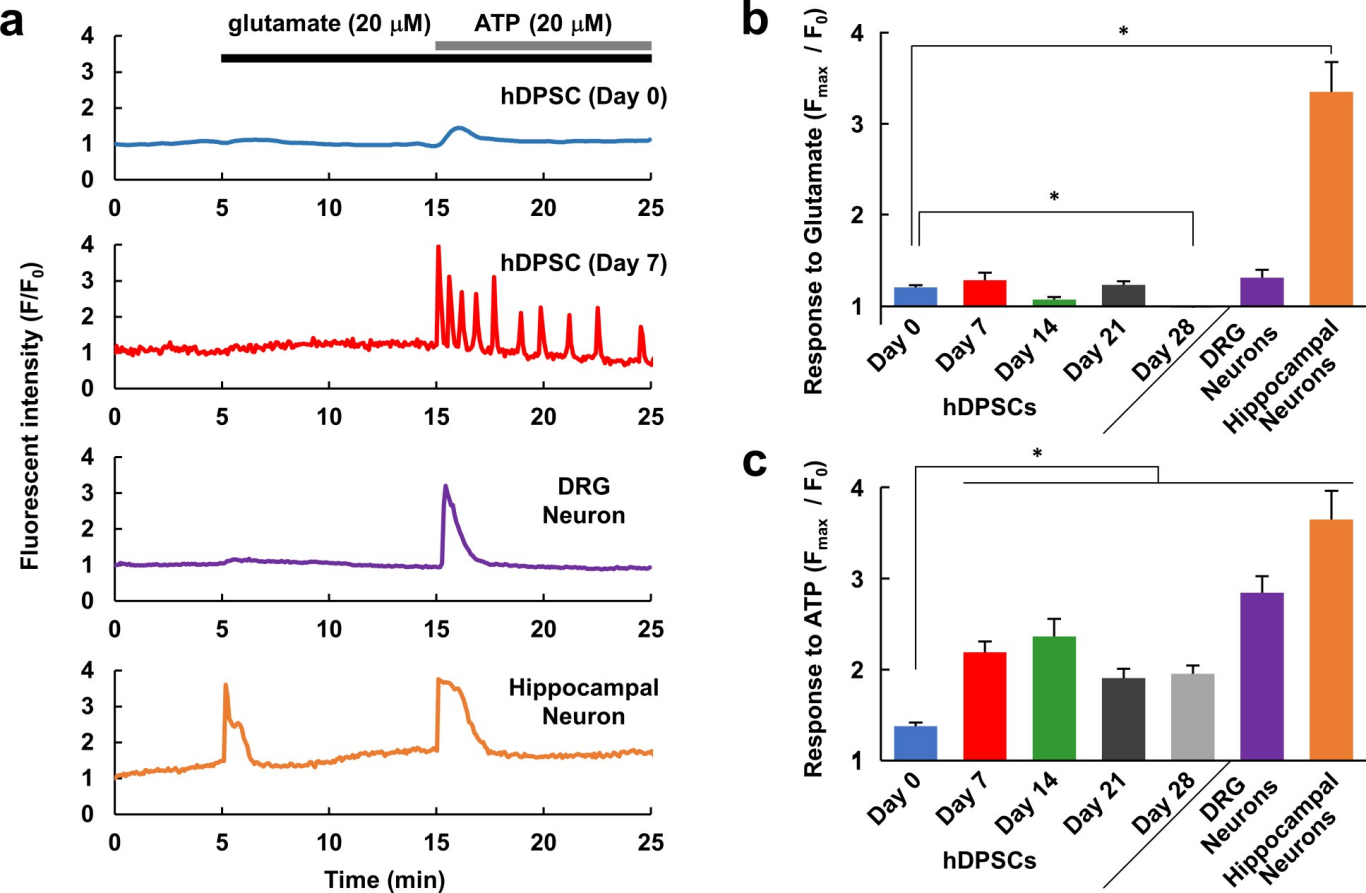
Ca^2+^ responses of hDPSCs, DRG and hippocampal neurons. (a) Representative Ca^2+^ responses to glutamate (20 μM) and ATP (20 μM) application in undifferentiated (Day 0) and differentiated (Day 7) hDPSCs and cultured rat DRG and hippocampal neurons. Comparison of the response amplitudes (F_max_/F_0_) to glutamate (b) and ATP (c) in hDPSCs differentiated for the indicated days, DRG neurons, and hippocampal neurons (n = 210, 84, 35, 78, 94, 43, or 33 cells, respectively, from 2–5 different experiments for each condition). Error bars, standard error of the mean. * *P* < 0.05 compared to Day 0.

To determine whether the differentiated hDPSCs would be responsive to extracellular signals derived from other neurons and glial cells, Ca^2+^ response to glutamate, the most abundant excitatory neurotransmitter in the CNS, and adenosine triphosphate (ATP), a major signal between neurons and glial cells, were observed. In addition, we evaluated which neuron responses the hDPSC responses resembled by comparing the responses to those of DRG and hippocampal neurons, which are types of peripheral and central neurons, respectively. None of the hDPSCs (Day 0–Day 28) exhibited more than a small response to glutamate (Fig 5A and 5B). Similarly, cultured rat DRG neurons exhibited only a small or no response to glutamate, whereas cultured rat hippocampal neurons produced a large increase in Ca^2+^ increase in response to glutamate application (Fig 5A). In contrast, while ATP elicited a small Ca^2+^ response in hDPSCs at Day 0, it elicited a large Ca^2+^ response at Day 7 and thereafter (Fig 5A and 5C). Similar to the differentiated hDPSCs, both DRG and hippocampal neurons exhibited a large Ca^2+^ response to ATP (Fig 5A and 5C). These data indicate that hDPSCs exhibit peripheral-neuron-like responses rather than CNS-neuron-like responses to glutamate and ATP.

**Fig 6:**
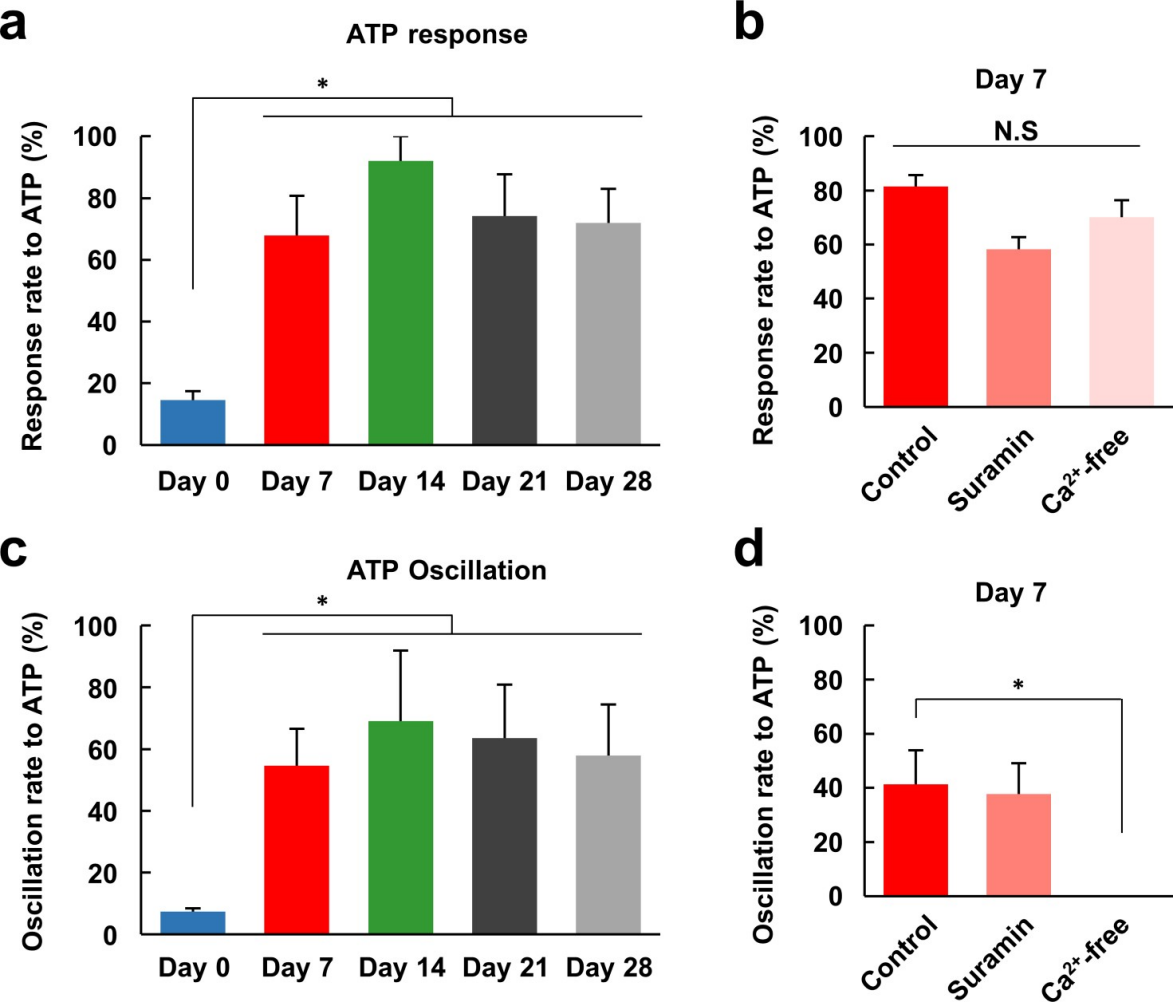
ATP-induced Ca^2+^ oscillation requires extracellular Ca^2+^. Comparison of the average percentage of responding cells (response rate) (a) and the average percentage of cells exhibiting Ca^2+^ oscillations (oscillation rate) (c) in response to ATP application in hDPSCs differentiated for the indicated days (n = 6, 5, 3, 3, or 2 cell populations for each condition, respectively). Response rate (b) and oscillation rate (d) upon ATP application in control conditions, in the presence of suramin (500 μM) or in Ca^2+^-free conditions for hDPSCs differentiated for seven days (n = 6, 3, or 3 cell populations for each condition, respectively). Error bars, standard error of the mean. * *P* < 0.05 compared to Day 0 or controls.

While approximately 20% of the undifferentiated hDPSCs exhibited a large response to ATP, after seven days of differentiation approximately 70% of the cells did so, and some showed oscillating Ca^2+^ increases (Fig 5A). Here, a large response was defined as F_max_/F_0_ > 1.5, and Ca^2+^ oscillation was defined as more than two Ca^2+^ transients in response to ATP application. The response rate to ATP was nearly saturated at Day7, and hDPSCs maintained this high level of responsiveness to ATP during further differentiation (Fig 6A). To examine the mechanism of the Ca^2+^ response to ATP, Ca^2+^ responses were observed in the presence of suramin, a substance with a broad spectrum of effects including inhibition of several types of P2 purinergic receptors, or in Ca^2+^-free medium. Neither suramin nor the Ca^2+^-free medium had a significant effect on the responsiveness of differentiated hDPSCs (Day 7) to ATP (Fig 6B), indicating that the ATP was received by a suramin-insensitive receptor that induced the release of Ca^2+^ from intracellular stores. In the differentiated hDPSCs, 40–70% of the cells exhibited Ca^2+^ oscillations in response to ATP application, whereas almost none of the undifferentiated cells showed this response (Fig 6C). Suramin had no effect on the oscillation rate (Fig 6D). In the Ca^2+^-free condition, although the transient increase in Ca^2+^ was not affected (Fig 6B), the Ca^2+^ oscillation that followed was completely abolished (Fig 6D). These data indicate that in the first transient response and in the following oscillations, Ca^2+^ is mobilized from different sources, and only the oscillations require extracellular Ca^2+^. From Figs 5 and 6, we concluded that the responses of hDPSCs to extracellular signals are fully differentiated in 7 days. Combined with the expression of peripheral neuron markers, hDPSCs differentiated for 14 days were considered as maturated peripheral neurons.

### Ca^2+^ response to TRP channel activation

**Fig 7:**
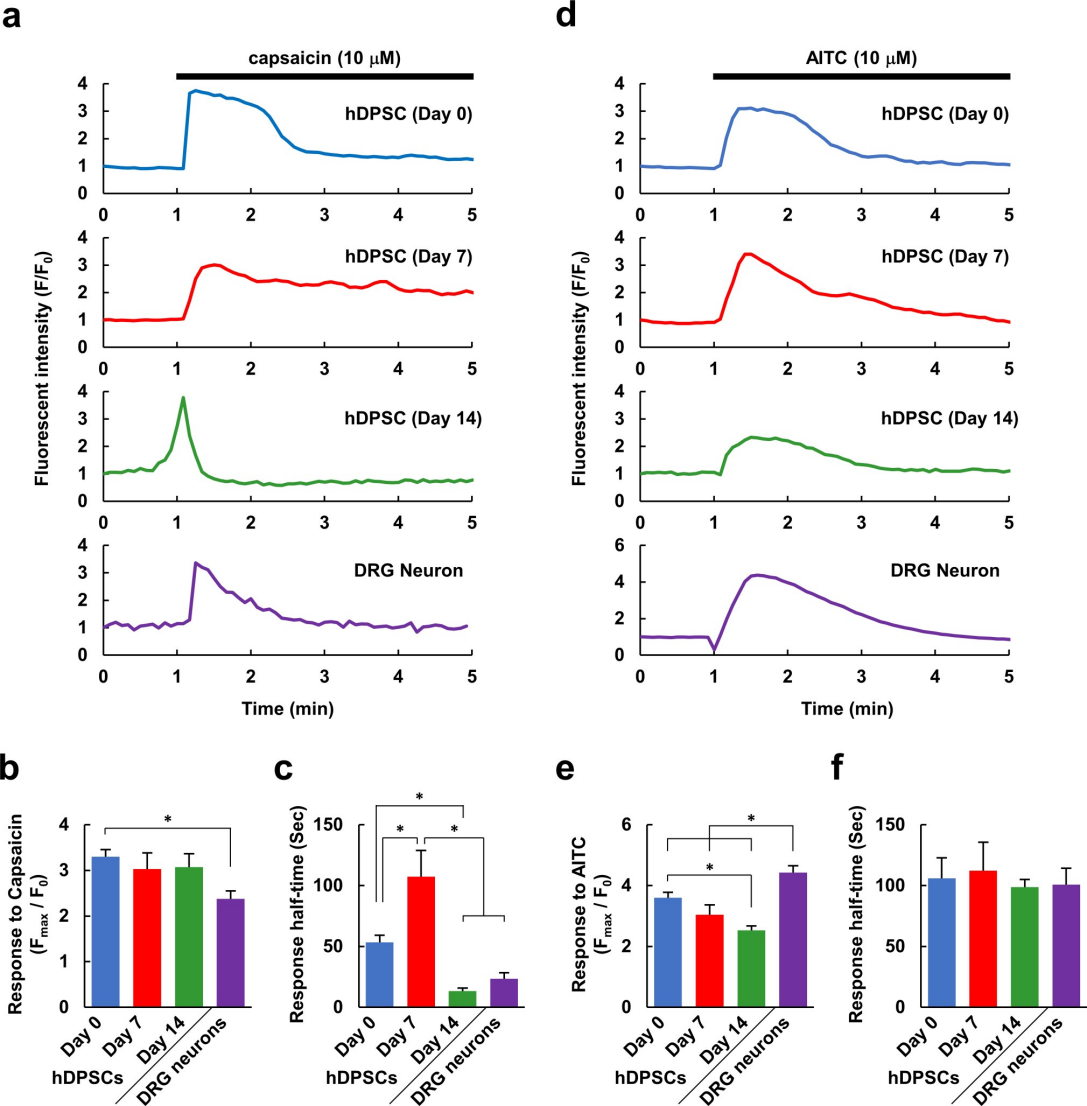
A transient receptor potential (TRP) channel agonist induces a Ca^2+^ response in hDPSCs. (a) Representative Ca^2+^ responses to capsaicin (10 μM), an activator of TRPV1 channels, in undifferentiated (Day 0) and differentiated (Day 7 and Day 14) hDPSCs and in cultured DRG neurons. (b) Amplitude of responses to capsaicin application in hDPSCs differentiated for the indicated days and in cultured DRG neurons (n = 92, 19, 51, or 23 cells, respectively, from 2–4 different experiments). (c) Response half-time of capsaicin-induced Ca^2+^ responses (n = 79, 16, 40, or 19 cells, respectively). (d) Representative Ca^2+^ responses to allyl isothiocyanate (AITC, 10 μM), an activator of TRPA1 channels, in hDPSCs differentiated for the indicated days and cultured DRG neurons. (e) Amplitude of responses to AITC application in hDPSCs differentiated for the indicated days and in cultured DRG neurons (n = 78, 13, 35, or 27 cells, respectively, from 2–4 different experiments). (f) Response half-time of AITC-induced Ca^2+^ responses (n = 68, 13, 32, or 25 cells, respectively). Error bars, standard error of the mean. * *P* < 0.05 in a comparison of all combinations in each graph.

To further test the hypothesis that differentiated hDPSCs would be useful as a model of human peripheral neurons, we observed Ca^2+^ responses to TRP channel activators. In general, TRP channels are characterized by responses to several types of physicochemical stimulation: temperature, osmotic pressure, the redox state of cells, and foreign chemical substances, such as capsaicin and menthol [46,47]. In particular, the TRPV1 and TRPA1 channels are important components of the pain reception system in the PNS and have attracted attention as drug targets for the treatment of pain [10,48,49]. We therefore examined Ca^2+^ responses to capsaicin, an agonist of the TRPV1 channel, and allyl isothiocyanate (AITC), an agonist of the TRPA1 channel. Since hSPSCs acquired the peripheral neuron-like properties within 14 days, the responses of hDPSCs to the TRP channel activators were observed on Day 14 and compared with the responses of DRG neurons. In addition, changes in response with differentiation were also investigated by comparing the responses on Day 14 with Day 0 and 7. Both capsaicin and AITC induced Ca^2+^ increases in undifferentiated (Day 0) and differentiated (Day 7 and Day 14) hDPSCs and DRG neurons, indicating that all of these cells expressed both types of TRP channel (Fig 7A and 7D). Although the amplitude of the response to capsaicin was similar in all the cell types, the response half-time was shorter in Day 14 hDPSCs, and their Ca^2+^ transients were sharper and more like those of DRG neurons (Fig 7A–7C). In contrast, while the half-time of the response to AITC treatment did not differ between cell types, the response amplitude decreased with increasing duration of differentiation (Fig 7D–7F). The shapes of the Ca^2+^ responses to both capsaicin and AITC in Day 14 hDPSCs were similar to those of cultured rat DRG neurons (Fig 7A and 7D).

## Discussion

In this study, we have demonstrated differentiation of hDPSCs toward neurons both in terms of their expression of marker proteins and in terms of their functions examined using Ca^2+^ imaging (Figs 1–4). In our experiments, βIII-tubulin-positive processes were observed in hDPSCs after seven days in differentiation media, and bipolar type neuron-like cells with elongated neurite-like processes were observed after fourteen days (Fig 1). Whereas differentiated and undifferentiated hDPSCs also expresses a neuronal stem cell marker, nestin, and an astrocyte marker, glial fibrillary acidic protein (GFAP) (S1 Fig and also reported from other laboratories [37,50]), we have demonstrated that the mRNA expression level of GFAP is much lower than that of βIII-tubulin in undifferentiated and differentiated hDPSC [51]. Therefore, βIII-tubulin predominates over GFAP in hDPSCs. Although the expression of nestin was detected in differentiated hDPSCs, expression levels of Brn-3a, a peripheral neuron marker, TRPV1, one of the nociceptors, and substance-P, a pain-mediating neurotransmitter, increased with differentiation and almost saturated on Day 14 (Fig 2). These indicate that hDPSCs differentiated toward peripheral neurons and that peripheral marker expressions matured to some extent within 14 days, although they are not yet mature neurons. The differentiated hDPSCs may sustain capability for further differentiation, at least, on Day 14 as the expression level of Brn-3a increased again on Day 28. To assess whether there was a correlation between the levels of marker expression and neuronal function, we observed the expression of marker proteins and Ca^2+^ responses to high concentrations of KCl in the same cells. The hDPSCs differentiated for 14 days increased all of Brn-3a and substance-P expression and Ca^2+^ response, while there was no difference in the distribution of expression levels between the no or small Ca^2+^ response group and the large Ca^2+^ response group (Fig 3). This suggests that hDPSCs differentiate in a different manner in the marker expression and in the function. We also confirmed an increase in voltage-gated Na^+^ channels in seven days differentiation using Ca^2+^ imaging (Fig 4). These data indicate that hDPSCs differentiate toward neurons, and also that cells with neuron-like protein expression patterns show neuron-like functions within 14 days of differentiation. To the best of our knowledge, this is the first report that demonstrates the differentiation of hDPSCs toward neuron-like cells in terms of both their marker protein expression and their function.

**Fig 8:**
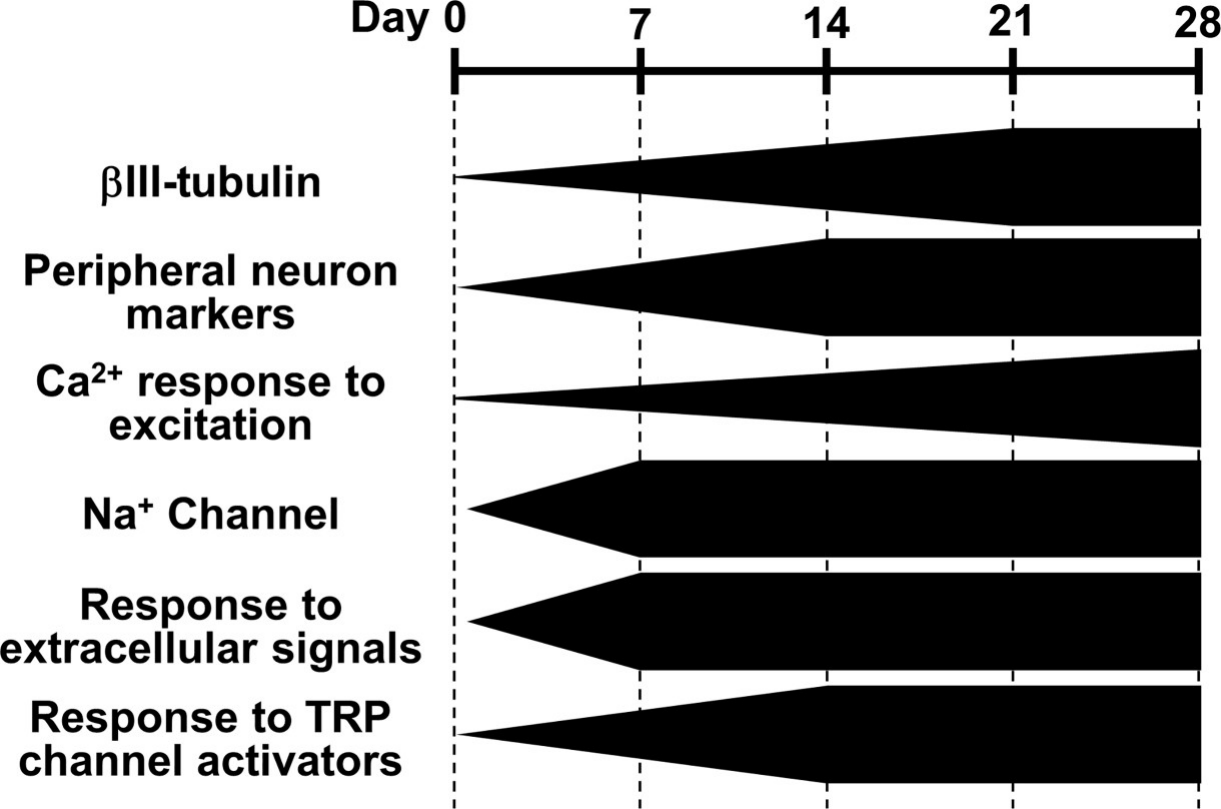
Summary of the differentiation of hDPSCs to peripheral neurons in each aspect revealed in this study.

Next, we evaluated the possibility of using hDPSCs as an *in vitro* cell model of peripheral neurons. Although they had differentiated toward neuron-like cells, they exhibited only small or no responses to glutamate, which is a major excitatory neurotransmitter in the CNS (Fig 5). Cultured rat DRG neurons also exhibited small or no increases in Ca^2+^ in response to glutamate, unlike cultured rat hippocampal neurons. On the other hand, whereas undifferentiated hDPSCs also only exhibited small responses to ATP, the differentiated hDPSCs exhibited large responses, similar to those of DRG and hippocampal neurons (Fig 5). This suggests that the hDPSCs differentiated in the medium we used behaved as peripheral neurons rather than central neurons, which makes sense given the peripheral-tissue origin of hDPSCs [21]. To further investigate the peripheral-neuron-like responses of hDPSCs, we analyzed their Ca^2+^ responses with respect to nociception. The Ca^2+^ increase evoked by ATP was not suppressed by suramin or Ca^2+^-free conditions (Fig 6), suggesting that ATP mobilizes Ca^2+^ from intracellular stores via metabotropic P2Y-type receptors. In peripheral neurons, Ca^2+^ is mobilized from the endoplasmic reticulum via the activation of P2Y receptors that are involved in nociception [52]. In addition, the agonists of the TRPV1 and TRPA1 channels elicited Ca^2+^ responses in both differentiated and undifferentiated hDPSCs, as well as in DRG neurons, and the shapes of the Ca^2+^ responses became more similar to those of DRG neurons over 14 days of differentiation (Fig 7). Although the expression level of TPRV1 channel in hDPSC was increased with differentiation (Fig 2), the amplitude of the response to capsaicin was not increased but it became transient in hDPSC (Fig 7A–7C). Considering that the response to KCl also became transient with differentiation (Fig 3A), hDPSCs would upregulate not only Ca^2+^ channels but also Ca^2+^ extrusion and storage mechanisms. Upregulation of plasma membrane Ca^2+^ ATPase (PMCA) activity, which accelerates Ca^2+^ extrusion from cytosol, is observed in the developing stage of brain and during synaptogenesis in neurons [53]. PMCA is one of the principal mechanism of Ca^2+^ extrusion in DRG neurons [54], and increased Ca^2+^ efflux via PMCA after application of capsaicin in cultured DRG neurons has been reported [55]. These reports indicate that not only the mechanism for increasing Ca^2+^ mobilization but also the one for Ca^2+^ clearance are important for neuronal development and maturation [54,56]. This alteration might be responsible for the transient Ca^2+^ responses without changing its amplitude in hDPSC, which turns Ca^2+^ increase into a sharp signal similar to that observed in DRG neurons (Fig 7). Also, this could be the reason why the Ca^2+^ responses were independent of marker expression (Fig 3). The amplitude and half-time of the Ca^2+^ response did not correlate with the expression of differentiation marker, probably due to the variety of the expression of Ca^2+^ mobilization and clearance mechanisms in each cell, even though averaged Ca^2+^ response became grater and sharper with neuronal differentiation. TRP channels are implicated in nociception, and in particular, TRPV1 and TRPA1 play important roles in the PNS [10,48,49]. Therefore, our results indicate that hDPSCs are a promising candidate for an *in vitro* cell model of peripheral neurons in experiments involving nociception and subsequent pain response. The neuronal maturation of differentiated hDPSCs in each aspect was summarized in Fig 8. To the best of our knowledge, this is the first report that demonstrates the peripheral neuron-like response of neuronally differentiated hDSPCs to extracellular stimuli.

In this study, we have demonstrated that differentiated hDPSCs are a candidate for *in vitro* modeling of human peripheral neurons. While hDPSCs have been used in fundamental studies and in regenerative medicine focused on neurodegenerative CNS diseases in animal models [38–41], our findings indicate that they will also be useful for studies of the PNS. While ES and iPS cells also have the potential to differentiate toward peripheral neurons [13,14], hDPSCs would become more convenient model cells. The relief of pain is an important aspect of improving quality of life. Using hDPSCs as an *in vitro* experimental model of pain sensation would provide a powerful tool for uncovering the mechanisms underlying human nociception.

## Supporting information

S1 Fig Immunofluorescence images of the GFAP and nestin in undifferentiated and differentiated human dental pulp stem cells (hDPSCs). Confocal immunofluorescence images showing GFAP (green) and nestin (white) of hDPSCs cultured in differentiation medium for 0, 7, 14, 21 or 28 days (Day 0, Day 7, Day 14, Day21 and Day28). Scale bar, 10 μm.
(TIF)

S1 Data (XLSX)

## References

[1] Kaliyaperumal S, Wilson K, Aeffner F, Dean C. Animal Models of Peripheral Pain: Biology Review and Application for Drug Discovery. Toxicol Pathol. 2020;48(1):202–19. 10.1177/0192623319857051 31269874

[2] Saito N, Shima R, Yamada Y, Nagaoka M, Ito E, Yoshioka T. A Proposed Molecular Mechanism for Physical Analgesia in Chronic Pain. Neural Plast. 2018;2018:1–8. 10.1155/2018/1260285 29887879 PMC5985137

[3] Lumpkin EA, Caterina MJ. Mechanisms of sensory transduction in the skin. Nature. 2007;445(7130):858–65. 10.1038/nature05662 17314972

[4] Ossipov MH. The Perception and Endogenous Modulation of Pain. Scientifica (Cairo). 2012;2012:1–25. 10.6064/2012/561761 24278716 PMC3820628

[5] Bell A. The neurobiology of acute pain. Vet J. 2018;237:55–62. 10.1016/j.tvjl.2018.05.004 30089546

[6] Alhadeff AL, Su Z, Hernandez E, Klima ML, Phillips SZ, Holland RA, et al. A Neural Circuit for the Suppression of Pain by a Competing Need State. Cell. 2018;173(1):140–152.e15. 10.1016/j.cell.2018.02.057 29570993 PMC5877408

[7] Kim YS, Anderson M, Park K, Zheng Q, Agarwal A, Gong C, et al. Coupled Activation of Primary Sensory Neurons Contributes to Chronic Pain. Neuron. 2016;91(5):1085–96. 10.1016/j.neuron.2016.07.044 27568517 PMC5017920

[8] Inquimbert P, Moll M, Latremoliere A, Tong C-K, Whang J, Sheehan GF, et al. NMDA Receptor Activation Underlies the Loss of Spinal Dorsal Horn Neurons and the Transition to Persistent Pain after Peripheral Nerve Injury. Cell Rep. 2018;23(9):2678–89. 10.1016/j.celrep.2018.04.107 29847798 PMC6276118

[9] Pinho-Ribeiro FA, Verri WA, Chiu IM. Nociceptor Sensory Neuron–Immune Interactions in Pain and Inflammation. Trends Immunol. 2017;38(1):5–19. 10.1016/j.it.2016.10.001 27793571 PMC5205568

[10] Moore C, Gupta R, Jordt S-E, Chen Y, Liedtke WB. Regulation of Pain and Itch by TRP Channels. Neurosci Bull. 2018;34(1):120–42. 10.1007/s12264-017-0200-8 29282613 PMC5799130

[11] Thomson JA. Embryonic Stem Cell Lines Derived from Human Blastocysts. Science (80-). 1998;282(5391):1145–7. 10.1126/science.282.5391.1145 9804556

[12] Takahashi K, Tanabe K, Ohnuki M, Narita M, Ichisaka T, Tomoda K, et al. Induction of Pluripotent Stem Cells from Adult Human Fibroblasts by Defined Factors. Cell. 2007;131(5):861–72. 10.1016/j.cell.2007.11.019 18035408

[13] Namer B, Schmidt D, Eberhardt E, Maroni M, Dorfmeister E, Kleggetveit IP, et al. Pain relief in a neuropathy patient by lacosamide: Proof of principle of clinical translation from patient-specific iPS cell-derived nociceptors. EBioMedicine. 2019;39:401–8. 10.1016/j.ebiom.2018.11.042 30503201 PMC6354557

[14] Eberhardt E, Havlicek S, Schmidt D, Link AS, Neacsu C, Kohl Z, et al. Pattern of Functional TTX-Resistant Sodium Channels Reveals a Developmental Stage of Human iPSC- and ESC-Derived Nociceptors. Stem Cell Reports. 2015;5(3):305–13. 10.1016/j.stemcr.2015.07.010 26321143 PMC4618592

[15] Iaquinta MR, Mazzoni E, Bononi I, Rotondo JC, Mazziotta C, Montesi M, et al. Adult Stem Cells for Bone Regeneration and Repair. Front Cell Dev Biol. 2019;7:268. 10.3389/fcell.2019.00268 31799249 PMC6863062

[16] Chen L, Qu J, Cheng T, Chen X, Xiang C. Menstrual blood-derived stem cells: toward therapeutic mechanisms, novel strategies, and future perspectives in the treatment of diseases. Stem Cell Res Ther. 2019;10(1):406. 10.1186/s13287-019-1503-7 31864423 PMC6925480

[17] Anitua E, Troya M, Zalduendo M. Progress in the use of dental pulp stem cells in regenerative medicine. Cytotherapy. 2018;20(4):479–98. 10.1016/j.jcyt.2017.12.011 29449086

[18] Yang X, Li L, Xiao L, Zhang D. Recycle the dental fairy’s package: overview of dental pulp stem cells. Stem Cell Res Ther. 2018;9(1):347. 10.1186/s13287-018-1094-8 30545418 PMC6293656

[19] Mehrotra P, Tseropoulos G, Bronner ME, Andreadis ST. Adult tissue–derived neural crest-like stem cells: Sources, regulatory networks, and translational potential. Stem Cells Transl Med. 2020;9(3):328–41. 10.1002/sctm.19-0173 31738018 PMC7031649

[20] Pisciotta A, Bertoni L, Vallarola A, Bertani G, Mecugni D, Carnevale G. Neural crest derived stem cells from dental pulp and tooth-associated stem cells for peripheral nerve regeneration. Neural Regen Res. 2020;15(3):373. 10.4103/1673-5374.266043 31571644 PMC6921350

[21] Gronthos S, Mankani M, Brahim J, Robey PG, Shi S. Postnatal human dental pulp stem cells (DPSCs) in vitro and invivo. Proc Natl Acad Sci. 2000;97(25):13625–30. 10.1073/pnas.240309797 11087820 PMC17626

[22] Mastrolia I, Foppiani EM, Murgia A, Candini O, Samarelli AV, Grisendi G, et al. Challenges in Clinical Development of Mesenchymal Stromal/Stem Cells: Concise Review. Stem Cells Transl Med. 2019;8(11):1135–48. 10.1002/sctm.19-0044 31313507 PMC6811694

[23] Potdar PD. Human dental pulp stem cells: Applications in future regenerative medicine. World J Stem Cells. 2015;7(5):839. 10.4252/wjsc.v7.i5.839 26131314 PMC4478630

[24] Chang C-C, Chang K-C, Tsai S-J, Chang H-H, Lin C-P. Neurogenic differentiation of dental pulp stem cells to neuron-like cells in dopaminergic and motor neuronal inductive media. J Formos Med Assoc. 2014;113(12):956–65. 10.1016/j.jfma.2014.09.003 25438878

[25] Tamaki Y, Nakahara T, Ishikawa H, Sato S. In vitro analysis of mesenchymal stem cells derived from human teeth and bone marrow. Odontology. 2013;101(2):121–32. 10.1007/s10266-012-0075-0 22772774

[26] Kanafi M, Majumdar D, Bhonde R, Gupta P, Datta I. Midbrain Cues Dictate Differentiation of Human Dental Pulp Stem Cells Towards Functional Dopaminergic Neurons. J Cell Physiol. 2014;229(10):1369–77. 10.1002/jcp.24570 24477667

[27] Ebrahimi B, Yaghoobi MM, Kamal-Abadi AM, Raoof M. Human dental pulp stem cells express many pluripotency regulators and differentiate into neuronal cells. Neural Regen Res. 2011;6(34):2666–72. 10.3969/j.issn.1673-5374.2011.34.004

[28] Ganapathy K, Datta I, Bhonde R. Astrocyte-Like Cells Differentiated from Dental Pulp Stem Cells Protect Dopaminergic Neurons Against 6-Hydroxydopamine Toxicity. Mol Neurobiol. 2019;56(6):4395–413. 10.1007/s12035-018-1367-3 30327976

[29] Shen W-C, Lai Y-C, Li L-H, Liao K, Lai H-C, Kao S-Y, et al. Methylation and PTEN activation in dental pulp mesenchymal stem cells promotes osteogenesis and reduces oncogenesis. Nat Commun. 2019;10(1):2226. 10.1038/s41467-019-10197-x 31110221 PMC6527698

[30] Wilson R, Urraca N, Skobowiat C, Hope KA, Miravalle L, Chamberlin R, et al. Assessment of the Tumorigenic Potential of Spontaneously Immortalized and hTERT -Immortalized Cultured Dental Pulp Stem Cells. Stem Cells Transl Med. 2015;4(8):905–12. 10.5966/sctm.2014-0196 26032749 PMC4511141

[31] Dunaway K, Goorha S, Matelski L, Urraca N, Lein PJ, Korf I, et al. Dental Pulp Stem Cells Model Early Life and Imprinted DNA Methylation Patterns. Stem Cells. 2017;35(4):981–8. 10.1002/stem.2563 28032673 PMC5367950

[32] Victor AK, Reiter LT. Dental pulp stem cells for the study of neurogenetic disorders. Hum Mol Genet. 2017;26(R2):R166–71. 10.1093/hmg/ddx208 28582499 PMC5886465

[33] Mortada I, Mortada R, Al Bazzal M. Dental pulp stem cells and the management of neurological diseases: An update. J Neurosci Res. 2018;96(2):265–72. 10.1002/jnr.24122 28736906

[34] Han Q, Wang Q, Wu J, Li M, Fang Y, Zhu H, et al. Nell-1 promotes the neural-like differentiation of dental pulp cells. Biochem Biophys Res Commun. 2019;513(2):515–21. 10.1016/j.bbrc.2019.04.028 30979495

[35] Arthur A, Rychkov G, Shi S, Koblar SA, Gronthos S. Adult Human Dental Pulp Stem Cells Differentiate Toward Functionally Active Neurons Under Appropriate Environmental Cues. Stem Cells. 2008;26(7):1787–95. 10.1634/stemcells.2007-0979 18499892

[36] Király M, Porcsalmy B, Pataki Á, Kádár K, Jelitai M, Molnár B, et al. Simultaneous PKC and cAMP activation induces differentiation of human dental pulp stem cells into functionally active neurons. Neurochem Int. 2009;55(5):323–32. 10.1016/j.neuint.2009.03.017 19576521

[37] Li D, Zou X-Y, El-Ayachi I, Romero LO, Yu Z, Iglesias-Linares A, et al. Human Dental Pulp Stem Cells and Gingival Mesenchymal Stem Cells Display Action Potential Capacity In Vitro after Neuronogenic Differentiation. Stem Cell Rev Reports. 2019;15(1):67–81. 10.1007/s12015-018-9854-5 30324358 PMC6358481

[38] Gnanasegaran N, Govindasamy V, Simon C, Gan QF, Vincent-Chong VK, Mani V, et al. Effect of dental pulp stem cells in MPTP-induced old-aged mice model. Eur J Clin Invest. 2017;47(6):403–14. 10.1111/eci.12753 28369799

[39] Zhang X, Zhou Y, Li H, Wang R, Yang D, Li B, et al. Transplanted Dental Pulp Stem Cells Migrate to Injured Area and Express Neural Markers in a Rat Model of Cerebral Ischemia. Cell Physiol Biochem. 2018;45(1):258–66. 10.1159/000486772 29402798

[40] Leong WK, Henshall TL, Arthur A, Kremer KL, Lewis MD, Helps SC, et al. Human Adult Dental Pulp Stem Cells Enhance Poststroke Functional Recovery Through Non-Neural Replacement Mechanisms. Stem Cells Transl Med. 2012;1(3):177–87. 10.5966/sctm.2011-0039 23197777 PMC3659845

[41] Wang F, Jia Y, Liu J, Zhai J, Cao N, Yue W, et al. Dental pulp stem cells promote regeneration of damaged neuron cells on the cellular model of Alzheimer’s disease. Cell Biol Int. 2017;41(6):639–50. 10.1002/cbin.10767 28328017

[42] Takahashi H, Ishikawa H, Tanaka A. Regenerative medicine for Parkinson’s disease using differentiated nerve cells derived from human buccal fat pad stem cells. Hum Cell. 2017;30(2):60–71. 10.1007/s13577-017-0160-3 28210976

[43] Mochizuki M, Nakahara T. Establishment of xenogeneic serum-free culture methods for handling human dental pulp stem cells using clinically oriented in-vitro and in-vivo conditions. Stem Cell Res Ther. 2018;9(1):25. 10.1186/s13287-017-0761-5 29394956 PMC5797401

[44] Mochizuki M, Sagara H, Nakahara T. Type I collagen facilitates safe and reliable expansion of human dental pulp stem cells in xenogeneic serum-free culture. Stem Cell Res Ther. 2020;11(1):267. 10.1186/s13287-020-01776-7 32660544 PMC7359624

[45] Yamanaka R, Shindo Y, Hotta K, Suzuki K, Oka K. GABA-Induced Intracellular Mg2+ Mobilization Integrates and Coordinates Cellular Information Processing for the Maturation of Neural Networks. Curr Biol. 2018;28(24):3984–3991.e5. 10.1016/j.cub.2018.10.044 30528584

[46] Nilius B, Owsianik G. The transient receptor potential family of ion channels. Genome Biol. 2011;12(3):218. 10.1186/gb-2011-12-3-218 21401968 PMC3129667

[47] Moran MM, Xu H, Clapham DE. TRP ion channels in the nervous system. Curr Opin Neurobiol. 2004;14(3):362–9. 10.1016/j.conb.2004.05.003 15194117

[48] Giorgi S, Nikolaeva-Koleva M, Alarcón-Alarcón D, Butrón L, González-Rodríguez S. Is TRPA1 Burning Down TRPV1 as Druggable Target for the Treatment of Chronic Pain? Int J Mol Sci. 2019;20(12):2906. 10.3390/ijms20122906 31197115 PMC6627658

[49] Gouin O’L, Herondelle K, Lebonvallet N, Le Gall-Ianotto C, Sakka M, Buhé V, et al. TRPV1 and TRPA1 in cutaneous neurogenic and chronic inflammation: pro-inflammatory response induced by their activation and their sensitization. Protein Cell. 2017;8(9):644–61. 10.1007/s13238-017-0395-5 28364279 PMC5563280

[50] Feng X, Lu X, Huang D, Xing J, Feng G, Jin G, et al. 3D Porous Chitosan Scaffolds Suit Survival and Neural Differentiation of Dental Pulp Stem Cells. Cell Mol Neurobiol. 2014;34(6):859–70. 10.1007/s10571-014-0063-8 24789753 PMC11488894

[51] Kogo Y, Seto C, Totani Y, Mochizuki M, Nakahara T, Oka K, et al. Rapid differentiation of human dental pulp stem cells to neuron-like cells by high K+ stimulation. Biophys Physicobiology. 2020;2020. 10.2142/biophysico.BSJ-2020023 33240740 PMC7671740

[52] Zhang X, Li G. P2Y receptors in neuropathic pain. Pharmacol Biochem Behav. 2019;186(August):172788. 10.1016/j.pbb.2019.172788 31494119

[53] Mata AM, Rosario S. Plasma membrane Ca 2+ -ATPases in the nervous system during development and ageing. World J Biol Chem. 2010;1(7):229–34. 10.4331/wjbc.v1.i7.229 21537478 PMC3083968

[54] Khariv V, Elkabes S. Contribution of Plasma Membrane Calcium ATPases to neuronal maladaptive responses: Focus on spinal nociceptive mechanisms and neurodegeneration. Neurosci Lett. 2018;663:60–5. 10.1016/j.neulet.2017.08.003 28780172

[55] Pottorf WJ, Thayer SA. Transient rise in intracellular calcium produces a long-lasting increase in plasma membrane calcium pump activity in rat sensory neurons. J Neurochem. 2002;83(4):1002–8. 10.1046/j.1471-4159.2002.01221.x 12421373

[56] Forostyak O, Forostyak S, Kortus S, Sykova E, Verkhratsky A, Dayanithi G. Physiology of Ca2+ signalling in stem cells of different origins and differentiation stages. Cell Calcium. 2016;59(2–3):57–66. 10.1016/j.ceca.2016.02.001 26905828

