# Supplementary figures and images for: Peripheral-neuron-like properties of differentiated human dental pulp stem cells (hDPSCs)
Source: PLoS One. 2021 May 6;16(5):e0251356. doi: 10.1371/journal.pone.0251356 (PMC8101759; doi:10.1371/journal.pone.0251356)

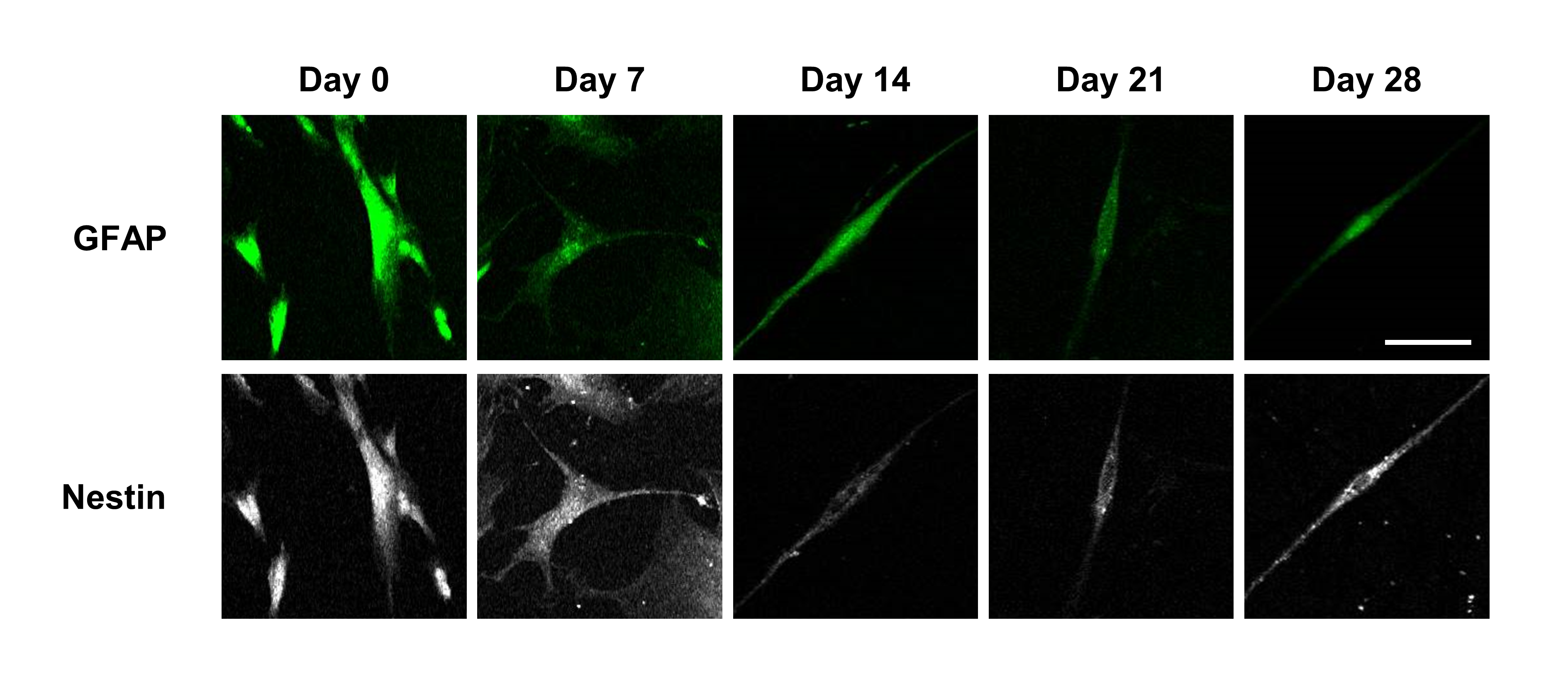

Supplement: S1 Fig — Confocal immunofluorescence images showing GFAP (green) and nestin (white) of hDPSCs cultured in differentiation medium for 0, 7, 14, 21 or 28 days (Day 0, Day 7, Day 14, Day21 and Day28). Scale bar, 10 μm. (TIF) [file pone.0251356.s001.tif]
